# Indicators of physical and emotional health in infants during hospitalization in paediatric cardiac intensive care units

**DOI:** 10.3389/fcvm.2026.1884496

**Published:** 2026-08-13

**Authors:** Hannah Ferentzi, Ralph C. A. Rippe, Stephan Schubert, Maika Böhm, Magdalena Blendermann, Katharina Riepen, Jos M. Latour, Felix Berger, Katharina R. L. Schmitt

**Affiliations:** 1 Developmental Paediatrics, Department of Congenital Heart Disease—Paediatric Cardiology, Deutsches Herzzentrum der Charité, Berlin, Germany; 2 Charité—Universitätsmedizin Berlin, Corporate Member of Freie Universität Berlin and Humboldt-Universität zu Berlin, Berlin, Germany; 3 Research Methods and Statistics, Institute of Education and Child Studies, Leiden University, Leiden, Netherlands; 4 Ruhr University Bochum, Heart and Diabetes Center NRW, Department of Pediatric Cardiology and Center for Congenital Heart Defects, Bad Oeynhausen, Germany; 5 University Bielefeld, Medical Faculty OWL, Heart and Diabetes Center NRW, Department of Pediatric Cardiology and Center for Congenital Heart Defects, Bad Oeynhausen, Germany; 6 School of Nursing and Midwifery, Faculty of Health, University of Plymouth, Plymouth, United Kingdom

**Keywords:** congenital heart disease, emotional functioning, family-centred care, infant mental health, infant physical health, paediatric cardiology, paediatric intensive care unit

## Abstract

**Introduction:**

Children with Congenital Heart Disease (CHD) are at risk for mental health difficulties. This study examines the relationship between parental satisfaction with Family-Centred Care (FCC), sociodemographic and medical variables, and infant physical and emotional health during hospitalization in Paediatric Intensive Care Units (PICUs).

**Methods:**

In this two-centre observational study, 305 families from two paediatric cardiac centres participated (52 fathers, 273 mothers, 23 couples). Infants were on average 120 days old (173 male, 132 female). Data were collected within 24 h after discharge from the PCICU. Questionnaires included a primary caregiver assessment, sociodemographic questionnaire, satisfaction with FCC (EMpowerment of PArents in The Intensive Care-30, EMPATHIC-30) questionnaire, and the Physical Health Summary Scale (PHSS) and Emotional Functioning Scale (EFS) of the Paediatric Quality of Life Inventory (PEDSQL) Infant Scales. Medical information was gathered from patient files.

**Results:**

Multilevel modelling revealed that higher EMPATHIC-30 scores on the Information subscale were linked to fewer physical symptoms. Infant sex, age, medical complexity, duration of PCICU stay, siblings, parental professional level, living distance from the centre, and parental role affected physical health scores. Higher scores on the EMPATHIC-30 subscale Care and Cure correlated with better emotional functioning, while higher scores on Professional Attitude were associated with lower emotional functioning. Infant age, prematurity, genetic comorbidity, PCICU duration, siblings, parental professional level, and migration background were associated with emotional functioning.

**Discussion:**

Findings indicate that unique combinations of medical and contextual factors are associated with physical and emotional health in infants, highlighting the need for tailored FCC interventions. This study was exploratory. Intervention studies are necessary to establish causality.

## 1 Introduction

Congenital heart disease (CHD) is the most common malformation among newborns, characterised by a malformation of the heart and/or intrathoracic vessels present at birth (1, 2). Children with CHD are at increased risk for mental health difficulties, including neurodevelopmental and emotional disorders (3). Early infancy is a developmental period with rapid physical growth, neuronal and sensory development, and the maturation of stress response systems. During this period, infants are particularly susceptible to environmental influences such as caregiving (4–6), nutrition (7), or socio-economic conditions (8). These early experiences have been shown to predict both physical and mental health outcomes later in life (9–12).

In Germany, nearly 90% of full hospital admissions of children with CHD for surgical or catheter interventions occur within the first 5 years of life, with approximately 80% taking place before the age of 12 months (13). The hospital environment exposes infants to a myriad of stressors, ranging from sensory overstimulation and restricted mobility to invasive procedures and neurotoxic medications, as well as disrupted parent-infant interactions. Hospitalization during infancy may be particularly challenging for infants with CHD due to complex neurodevelopmental vulnerabilities (14, 15). While our understanding of neurodevelopmental outcomes in infants with CHD is still limited, there are studies pointing towards increased vulnerability to environmental stressors, such as those encountered in Paediatric Cardiac Intensive Care Units (PICIU; 16–19). They contribute to lasting physiological changes and altered brain structure and function, ultimately resulting in cognitive, emotional and social difficulties later in life (20, 21). Capturing infants' well-being poses a challenge, however. While infants cannot communicate their needs verbally, experts agree that potential bias in the measurement of infants' quality of life can be minimized through proxy reports from the primary caregiver and completed on their child's behalf (22). Additional more objective reports from medical staff can further help capture infants' well-being in an unbiased manner. Hence, quantifying infants' difficulties and environmental factors using appropriate instruments is essential.

Modifying the hospital environment may be an effective strategy to improve mental health outcomes in this population. Family-Centred Care (FCC) is a holistic approach to facilitating a developmentally supportive environment, with care interventions on different levels (e.g., kangaroo care, interdisciplinary care rounds) sharing the common goal of meeting infant needs, as well as family needs and values during hospitalization (23). In neonatology, FCC interventions improve infant physical health outcomes [e.g., weight gain, readmission rates, (24, 25)], sleep and feeding (26), neurobehavioral outcomes (25), and parental outcomes [e.g., parental satisfaction with care, mental health outcomes, caregiving behaviors, (27–29)]. While empirical insights on FCC and infant mental health in neonatology are scarce, recent publications emphasize the necessity to investigate infant mental health as an important outcome in neonatology (30, 31).

FCC may also benefit infants with CHD, although empirical studies on this topic remain limited (32–35). One study points towards an FCC intervention (kangaroo care) improving attachment between mothers and infants with CHD (34), while another study showed positive effects of a similar intervention in children after cardiac surgery on vital signs (such as heart rate, systolic arterial pressure) and pain scores, indicating enhanced autonomic regulation (36). Both infant attachment and autonomic regulation are associated with emotional development, thus underscoring the centrality of relationship-based care, a core component of FCC, for infant emotional health (37).

Gaining insights on the role of FCC in the context of other environmental and medical factors for infants' physical and emotional health can guide our efforts towards the development of evidence-based interventions. Therefore, we aimed to explore infant physical and emotional health during hospitalization in the PCICU in a two-centre observational study. We investigated parental satisfaction with FCC as an indicator of infant physical and emotional health, next to socio-demographic and medical variables.

## 2 Methods

For a detailed description of our study methods and specific data preparation (e.g., recoding of variables), see published study protocol (38) and our findings on parental distress after PCICU stay (Ferentzi et al., under review).

### 2.1 Study design and participants

In this prospective, two-centre observational study, families of infants aged 0–12 months with CHD hospitalized in the PCICU at two specialized paediatric cardiac centres in Germany (Bad Oeynhausen = centre 1, Berlin = centre 2) were eligible. Participation was voluntary. Data were collected within 24 h after PCICU discharge.

### 2.2 Instruments

#### 2.2.1 Medical variables

We classified CHD diagnoses into three categories [complex, moderate and mild, (39)]. Cardiac disease complexity was rated using the Aristotle Comprehensive Complexity (ACC) score, with a higher score indicating greater complexity (40). Other medical variables included infant characteristics (age, sex), genetic comorbidities, prematurity, and duration of stay at PCICU in days.

#### 2.2.2 Primary caregiver assessment

We assessed primary caregiver status with a set of three questions (most caregiving responsibilities in the past month, amount of time spent with child in the past month, main visitor at the PCICU during the past week). Answer categories for all three questions were (single choice): “mainly father”, “mainly mother”, “both equally”.

#### 2.2.3 Socio-Demographic information

We used a purpose-designed questionnaire based on German demographic standards (41), comprising questions about the parental role (mother or father), birth year, migration background, first and second languages, educational and professional background, employment status, legal family status, living with partner, other children, and situational variables (room size PCICU—nights in single room divided by total number of nights; rooming-in—nights of parental overnight stay divided by total number of nights; living distance from centre).

#### 2.2.4 Parental satisfaction with FCC

We used the German version of the EMpowerment of PArents in The Intensive Care-30 Questionnaire [Lippold et al., unpublished manuscript; (42, 43)]. It contains five domains: Information, Care and Cure, Parental Participation, Organization and Professional Attitude. Responses range on a six-point scale, with a higher score indicating higher satisfaction with FCC. Internal reliability for the full-scale score is estimated at a Cronbach's alpha of 0.95 at paediatric cardiac care units (43) and a Cronbach's alpha of 0.97 as well as moderate to strong construct validity at neonatal intensive care units (44).

#### 2.2.5 Infant physical and emotional health

We used the German Version of the Paediatric Quality of Life Inventory (PEDSQL) Infant Scales (acute version) to assess infant physical and emotional health as rated by the parents (45). It assesses health-related quality of life in infants and is particularly suitable for hospital environments (46). The questionnaire consists of 36 items and assesses infant health within the past seven days and comprises five subscales (physical functioning, physical symptoms, emotional functioning, social functioning and cognitive functioning). For the purpose of this study, we used the Physical Health Summary Score (PHSS) comprising the subscales physical functioning (six items) and physical symptoms (10 items) to assess infant physical health. We used the Emotional Functioning Subscale (EFS, 12 items) to assess infant emotional health. Responses are given on a five-point scale ranging from 0 (“never”) to 4 (“almost always”), with a higher score indicating lower physical health, or more physical symptoms, and lower emotional functioning, or more emotional symptoms, respectively.

#### 2.2.6 Other

Parental emotional distress was assessed with the Depression Anxiety Stress Scale [DASS-21, (47, 48)], PCICU-related parental stress was assessed with the PSS:NICU-2 (49) and Unit adherence to FCC Practices was assessed with the Gap Analysis Tool provided by the Society of Critical Care Medicine (SCCM) (50). Results using these instruments were published in Ferentzi et al. (under review) and are beyond the scope of the current manuscript.

### 2.3 Procedure

We collected data from January 2021 to October 2022 and recruited families at discharge from PCICU. Oral information was provided and written informed consent was obtained from all parents with custodial rights. Questionnaires were handed out to the main visitor of the child in the past week instead of to the main caregiver as planned according to protocol, due to restricted family access to the PCICU during the COVID-19 pandemic. If both parents visited the child equally often, the questionnaire was handed to the primary caregiver. Parents completed the questionnaire within 24 h post PCICU discharge and returned the questionnaires in a sealed envelope.

### 2.4 Data analysis

We conducted descriptive analysis and preliminary analyses, particularly Missing Completely At Random (MCAR) analysis and nonlinear optimal scaling for questionnaire responses. In our main analyses, we investigated indicators of both infant physical and emotional health. We analysed associations between independent variables (EMPATHIC-30 scores, sociodemographic variables including situational factors, medical variables including infant characteristics), and two dependent variables (the subscales PHSS and EFS of the PEDSQL, respectively), adjusting explicitly for nesting within PCICU (centre 1, centre 2) at level two. Categorical variables were dummy-coded, using the lowest level as the reference level. The variable rooming-in was excluded from analyses, as only four parents reported rooming-in. For each of the dependent variables (PHSS, EFS), we estimated a series of multilevel models with incremental complexity. As Least Squares Estimation cannot be straightforwardly used for nested data (51), all models were estimated using Restricted Maximum Likelihood, defining the covariance structure through variance components, thus reducing estimation bias compared to standard Maximum Likelihood (52), and using the Huber- White Sandwich Estimator for the standard errors (53, 54). A Bayesian approach would also be estimated through maximum likelihood optimisation but requires an informed prior—uninformed or wrong priors can do much harm (55). However, due to the exploratory nature of the current study, such information is not available.

In the following, the model building steps for child physical and emotional health, respectively, are outlined. Model 5 represents our primary model to contain the (level of) associations and complexity of our main interest, with subsequent models reflecting exploratory model extensions.

1. Model 1: Unconditional means model
2. Model 2: Conditional means model (fixed effects with fixed slope association) for EMPATHIC-30 full-scale scores, adjusting for mean differences through a random intercept.
3. Model 3: Model 2, with added sociodemographic and medical variables, EMPATHIC-30 full-scale scores
4. Model 4: Model 3, with added random effect for the EMPATHIC-30 full-scale scores.
5. Model 5 (primary model): Model 3, with added backward selection to avoid overfitting, EMPATHIC-30 full-scale scores
6. Model 6: Model 3 with added backward selection, EMPATHIC-30 subscale scores instead of full-scale scores
7. Model 7: Model 5, additionally with all filters selected
8. Model 8 (final, best fitting model): Model 6, additionally with all filters selected

Filters to account for methodological challenges in our sample were: questionnaire filled out within 24 h after discharge from PCICU (no), dyadic response to questionnaires, i.e., questionnaire was filled out by both parents together (yes) and genetic comorbidity of child (status unclear), as well as PCICU stay (<7 days). Finally, we assessed non-linear associations between EMPATHIC-30 scores and dependent variables.

### 2.5 Ethics statement

Ethical approval was obtained from Charité Universitätsmedizin Berlin (EA2/032/20) and Ruhr-University Bochum (2020-713). The study was registered in the German Registry for Clinical Trials (Deutsches Register Klinischer Studien, DRKS), Trial Registration Number: DRKS00023964.

## 3 Results

### 3.1 Descriptive statistics

A total of 305 parents participated (*n* = 149 in centre 1, *n* = 156 in centre 2; 29 fathers, 250 mothers, 23 couples and for *n* = 3, this information is missing). In 157 cases, the main visitor in the past seven days was the mother, in eight cases the father, in 128 cases both, with missing information in 12 cases. In 278 cases, the primary caregiver filled out the questionnaire (including dyadic responses). The average age of the infants was *m* = 120.40 days (SD = 92.55), the average age of the parents was 32.08 (SD = 9.29) years. We found no significant differences in EMPATHIC-30 full-scale scores between centres [*t* (254.71) = −0.176, *p* = .860]. For an overview of the descriptive statistics, see Table 1.

**Table 1:** Descriptive statistics of full sample for both centres.

Variable (level coding)	Both centres *N* = 305	Centre 1 *n* = 149	Centre 2 *n* = 156	Test statistic for center differences
Categorical variables *N* (%)				
Child sex				***χ*^2^ (1) = 4.344** *
Male (0)	173 (56.7%)	75 (50.3%)	98 (62.8%)	
Female (1)	132 (43.3%)	74 (49.7%)	58 (37.2%)	
Siblings				χ^2^ (1) = 0.124
Yes (1)	172 (56.4%)	82 (55.0%)	90 (57.7%)	
No (0)	133 (43.6%)	67 (45.0%)	66 (42.3%)	
Prematurity				χ^2^ (1) = 0.007
Yes (1)	38 (12.5%)	18 (12.1%)	20 (12.8%)	
No (0)	264 (86.5%)	132 (87.9%)	133 (85.3%)	
Missing	3 (1.0%)	0 (0.0%)	3 (1.9%)	
Diagnosed genetic comorbidity child				χ^2^ (1) = 0.311
Yes (1)	54 (17.7%)	29 (19.5%)	25 (16.0%)	
No (0)	248 (81.3%)	120 (80.5%)	128 (82.1%)	
Missing	3 (1.0%)	0 (0.0%)	3 (1.9%)	
Warnes classification				**T(290) = 2.096** *
Mild (1)	33 (10.8%)	17 (11.4%)	16 (10.3%)	
Moderate (2)	121 (39.7%)	56 (37.6%)	65 (41.7%)	
Complex (3)	115 (37.7%)	52 (34.9%)	63 (40.4%)	
Other (0)	36 (11.8%)	24 (16.1%)	12 (7.7%)	
Legal status				**χ^2^ (2) = 26.382** ***
Married (1)	202 (66.2%)	119 (79.9%)	83 (53.2%)	
Single (3)	97 (31.8%)	30 (20.1%)	67 (42.9%)	
Divorced (4)	6 (2.0%)	0 (0.0%)	6 (3.8%)	
Partnership (living together)				χ^2^ (1) = 0.000
Yes (1)	253 (83.0%)	118 (79.2%)	135 (86.5%)	
No (0)	26 (8.5%)	12 (8.1%)	14 (9.0%)	
Missing	26 (8.5%)	19 (12.8%)	7 (4.5%)	
Professional level				χ^2^ (4) = 5.783
Unskilled/ semi-skilled (1)	11 (3.6%)	3 (2,0%)	8 (5.2%)	
Specialised (2)	30 (9.9%)	12 (8,1%)	18 (11.7%)	
Complex specialised (3)	171 (56.6%)	93 (62.8%)	78 50.6%)	
Highly complex specialised (4)	36 (11.9%)	16 10.8%)	20 (13.0%)	
Other (5)	54 (17.9%)	24 (16.2%)	30 (19.5%)	
Missing	3 (0.9%)	2 (1.3%)	1 (0.6%)	
School education				**χ^2^ (2) = 5.811** †
No/ unknown degree (1)	30 (9,9%)	9 (6.1%)	21 (13.6%)	
Middle to high education (2)	133 (44.2%)	72 (48.9%)	61 (39.6%)	
High education (3)	138 (45.8%)	66 (44.9%)	72 (46.8%)	
Missing	4 (1.3%)	1 (0.7%)	3 (1.3%)	
Occupational situation				χ^2^ (4) = 3.213
Full-time job (1)	92 (30.2%)	39 (26.2%)	53 (34.0%)	
Part-time job (2)	41 (13.4%)	20 (13.4%)	21 (13.5%)	
Marginal employment (4)	3 (1.0%)	1 (0.7%)	2 (1.3%)	
On (maternity/paternity) leave (10)	146 (47.9%)	77 (51.7%)	69 (44.2%)	
Not employed (11)	13 (4.3%)	5 (3.4%)	8 (5.1%)	
Missing	10 (3.3%)	7 (4.7%)	3 (1.9%)	
Mother tongue				**χ^2^ (3) = 6.602** †
German first, no second (1)	232 (76.1%)	112 (75.2%)	120 (76.9%)	
German first, second other (2)	47 (15.4%)	29 (19.5%)	18 (11.6%)	
Other first, second German (3)	22 (7.2%)	7 (4.7%)	15 (9.6%)	
Other first, no second (4)	4 (1.3%)	1 (0.6%)	3 (1.9%)	
Migration background child				χ^2^ (2) = 3.447
Third generation + (0)	192 (62.9%)	86 (57.7%)	106 (68.0%)	
Second generation (1)	111 (36.4%)	62 (41.6%)	49 (31.4%)	
First generation (2)	2 (0.7%)	1 (0.7%)	1 (0.6%)	
EMPATHIC-30 m (SD)				
Full-scale score	5.27 (0.67)	5.29 (0.61)	5.23 (0.74)	T(252) = −0.095
Subscale information	5.23 (0.79)	5.19 (0.80)	5.28 (0.79)	T(288) = 0.920
Subscale care and cure	5.26 (0.77)	5.21 (0.76)	5.32 (0.78)	T(275) = 1.474
Subscale Par. participation	4.97 (0.93)	5.08 (0.81)	4.86 (1.03)	**T(277) =** −**1.810**†
Subscale organisation	5.44 (0.61)	5.46 (0.56)	5.42 (0.66)	T(289) = −0.100
Subscale Prof. attitude	5.49 (0.65)	5.50 (0.59)	5.47 (0.70)	T(286*)* = 0.077
PEDSQL m (SD)				
Total score	1.33 (0.65)	1.49 (0.65)	1.18 (0.62)	**T(145.64) = −2.942** **
Physical health summary score (PHSS)	1.55 (0.81)	1.58 (0.69)	1.52 (0.89)	T(276) = −0.919
Physical functioning	2.17 (1.08)	2.13 (0.90)	2.20 (1.22)	T(199.67) = −0.481
Physical symptoms	1.02 (0.64)	1.34 (0.63)	0.92 (0.63)	**T(216.95) = −2.590** *
Emotional functioning subscale (EFS)	1.23 (0.86)	1.48 (0.88)	1.02 (0.78)	**T(169.77) = −3.763** ***
Continuous variables m (SD)				
Child age in days, *n* = 303	120.40 (92.55)	126.48 (100.00)	114.51 (84.64)	T(290) = −1.124
Living distance from centre, *n* = 293	94.88 (124.28)	105.75 (113.66)	84.22 (133.41)	T(286) = −1.488
Participant age in years, *n* = 265	32.08 (9.29)	32.15 (5.35)	32.01 (11.94)	(187) = −0.123
PCICU stay in days, *n* = 305	7.58 (8.10)	10.43 (8.95)	4.85 (6.06)	**T(259) =** −6.342***
Room size PICU, *n* = 305 *(Proportion of nights in single room/total number of nights)*	0.10 (0.29)	0.06 (0.20)	0.15 (0.34)	**T(251) = 3.008** **
Aristotle complexity score, *n* = 289	10.40 (3.55)	10.41 (3.73)	10.39 (3.41)	T(272) = −0.050

*Note.* Descriptive statistics are reported in N (%) for categorical variables and M (SD) for continuous variables. M, mean, SD, standard deviation. Total number of respondents vary because of missing data. Statistical significance (based on T-tests for independent samples and Chi-Square Tests) indicated by asterisks.

Bold indicates significant effect.

*** *p* < 0.001.

** *p* < 0.01.

* *p* < .05.

† *p* < 0.10.

Data on 23 couples with unclear main responder (dyadic response) were only used in the non-filtered models and were excluded in the filtered models to improve methodological precision. Accordingly, we effectively used *n* = 302 (29 fathers, 250 mothers, 23 couples) in the unfiltered models, and *n* = 279 (29 fathers, 250 mothers) in the data in the final models based on complete case selection and full filtering. For details on the filter variables, see Table 2. Table 3 provides a descriptive overview of the subsample (*n* = 57) after filters. This fully filtered subset shows similar descriptive characteristics compared to the total sample reported in Table 1.

**Table 2:** Filter variables.

Variable (level coding)	Both centres *N* = 305	Centre 1 *n* = 149	Centre 2 *n* = 156	Test statistic for center differences
Filter variables				
Suspected genetic comorbidity child				χ^2^ (1) = 4.49*
Yes (0)	6 (1.97%)	6 (4.03%)	0 (0.00%)	
No (1)	299 (98.03%)	143 (95.97%)	156 (100.00%)	
Response within 24 h				**χ^2^ (1) = 15.818** ***
Yes (1)	227 (74.4%)	128 (85.9%)	99 (63.5%)	
No (0)	68 (22.3%)	19 (12.8%)	49 (31.4%)	
Missing	10 (3.3%)	2 (1.3%)	8 (5.1%)	
Questionnaire respondent				χ^2^ (2) = 4.384
Both (2)	23 (7.5%)	15 (10.1%)	8 (5.1%)	
Mother (1)	250 (82.0%)	115 (77.2%)	135 (86.5%)	
Father (0)	29 (9.5%)	17 (11.4%)	12 (7.7%)	
PCICU-Stay ≥ 7 days				**χ^2^ (1) = 74.867** ***
Yes (1)	137 (44.9%)	105 (70.5%)	32 (20.5%)	
No (0)	168 (55.1%)	44 (29.5%)	124 (79.5%)	
Missing	0	0	0	

*Note.* Descriptive statistics are reported in N (%) for categorical variables. Total number of respondents vary because of missing data. Statistical significance (based on Chi-Square Tests) indicated by asterisks.

Bold indicates significant effect.

*** *p* < 0.001.

* *p* < .05.

**Table 3:** Descriptive statistics of subsample for both centres.

Variable (level coding)	Both centres *N* = 57
Categorical variables N (%)	
Child sex	
Male (0)	34 (60%)
Female (1)	23 (40%)
Siblings	
Yes (1)	35 (61%)
No (0)	22 (39%)
Prematurity	
Yes (1)	6 (10.5%)
No (0)	51 (89.5%)
Diagnosed genetic comorbidity child	
Yes (1)	14 (24.6%)
No (0)	43 (75.4%)
Warnes classification	
Mild (1)	2 (3.5%)
Moderate (2)	24 (42.1%)
Complex (3)	18 (31.6%)
Other (0)	13 (22.8%)
Legal status	
Married (1)	35 (61.4%)
Single (3)	21 (36.8%)
Divorced (4)	1 (1.8%)
Partnership (living together)	
Yes (1)	50 (88%)
No (0)	7 (22%)
Professional level	
Unskilled/ semi-skilled (1)	1 (1.8%)
Specialized (2)	7 (12.3%)
Complex specialized (3)	32 (56.1%)
Highly complex specialized (4)	9 (15.8%)
Other (5)	8 (14.0%)
School education	
No/ unknown degree (1)	5 (8.8%)
Middle to high education (2)	27 (47.4%)
High education (3)	25 (43.9%)
Occupational situation	
Full-time job (1)	13 (22.8%)
Part-time job (2)	9 (15.8%)
Marginal employment (4)	2 (3.5%)
On (maternity/paternity) leave (10)	31 (54.4%)
Not employed (11)	2 (3.5%)
Mother tongue	
German first, no second (1)	46 (80.7%)
German first, second other (2)	8 (14.0%)
Other first, second German (3)	3 (5.3%)
Other first, no second (4)	0
Migration background child	
Third generation+(0)	36 (63.2%)
Second generation (1)	20 (35.1%)
First generation (2)	1 (1.8%)
EMPATHIC-30 m (SD)	
Full-scale score	5.22 (0.80)
Subscale information	5.25 (0.87)
Subscale care and cure	5.21 (0.83)
Subscale Par. participation	4.99 (1.00)
Subscale organization	5.33 (0.79)
Subscale Prof. attitude	5.43 (0.74)
PEDSQL m (SD)	
Total score	1.37 (0.62)
Physical health summary score (PHSS)	1.67 (0.68)
Physical functioning	2.19 (0.89)
Physical symptoms	1.08 (0.65)
Emotional functioning subscale (EFS)	1.21 (0.81)
Continuous variables m (SD)	
Child age in days, *n* = 57	135.14 (90.15)
Living distance from centre, *n* = 57	77.89 (64.10)
Participant age in years, *n* = 57	30.72 (7.51)
PCICU stay in days, *n* = 57	10.61 (5.15)
Room size PICU, *n* = 57 *(Proportion of nights in single room/total number of nights)*	0.17 (0.35)
Aristotle complexity score, *n* = 57	11.14 (3.67)

### 3.2 Preliminary analyses

We assessed the nature of missing data using Little's MCAR test to check for bias risk in our main analysis. The test was not significant for any of the PEDSQL domains, over centres as well as per centre, and thus revealed no risk of bias using complete case analysis. The EMPATHIC-30 showed a significant MCAR test, *χ*^2^(46) = 609.0, *p* < .001, for centre 1, only. Given non-significant MCAR tests and a low percentage of missing observations (0.9%–8.5%), complete case analyses were performed, which is deemed sufficiently robust without the need for data imputation (56). An overview of the number of missing values per variable can be found in Table 1. A non-linear Principal Component Analysis revealed similar quantifications for the EMPATHIC-30 score 0 (not applicable) and score 6. The nonapplicable categories in the EMPATHIC-30 were therefore recoded to the highest value (6) for each item. More details on missing values analysis and recoding are provided in Ferentzi et al. (under review).

### 3.3 Main analyses

The above-mentioned models with incremental complexity are reported both for PHSS and EFS, starting with the model of primary interest, model 5. Statistical values and direction of effects are presented in detail for the primary model 5 and final model 8 only. None of the models revealed nonlinear associations, while all other association remained unchanged. Non-linear models are thus not reported. See Supplementary Tables S1, 2 for a detailed overview of statistical results of all consecutive model building steps.

#### 3.3.1 Infant physical health (PHSS)

The final, best fitting model is reported last. See Supplementary Table S3 for an overview of all fixed effects per model.

1. Model 5 (primary model)
  In model 5, the intercept was significant [*B* = 3.32, SE = 0.57, *t*(148) = 5.85, *p* < .001]. Empathic-30 full-scale scores were significant [*B* = −0.25, SE = 0.09, *t*(148) = −2.93, *p* = .004], with a higher score on the EMPATHIC-30 associated with a lower score on the PHSS, and thus fewer physical symptoms. The following fixed effects were significant: highly complex specialised professions [*B* = 0.87, SE = 0.24, *t*(148) = 3.63, *p* < .001], in that infants of caregivers with highly complex specialised professions scored higher than infants of caregivers with unskilled/semi-skilled professions. Migration background [*B* = −1.25, SE = 0.51, *t*(148) = −2.47, *p* = .015], in that infants of caregivers with first generation migration background scored lower on the PHSS than infants of caregivers with no migration background. Furthermore, caregiver age was significant in this model [*B* = −0.02, SE = 0.01, *t*(148) = −2.51, *p* = .013], reflecting fewer physical symptoms with increasing age of the parent. Finally, room size PCICU was significant [*B* = 0.36, SE = 0.18, *t*(148) = 2.01, *p* = .046], with a higher proportion of nights spent in a single room on the PCICU being associated with higher PHSS. This model explained approximately 16% of the variance (adjusted *R*^2^ = .16).
2. Model 6
  Model 6 revealed a significant intercept and showed that only the subscale EMPATHIC- Information was significant. Furthermore, similar to model 5, caregiver age was significant, as well as the fixed effect of highly complex specialised professions. The effect of a dyadic response to the questionnaire became significant in this model. This model explained approximately 16% of the variance (adjusted *R*^2^ = .16). For complete reporting of all statistical effects of model 6, see Supplementary Table 1.
  Next, we repeated models 5 and 6 for filtered cases only to obtain a subsample that mitigated methodological challenges as defined above.
3. Model 7
  In model 7, the intercept was not significant. Highly complex specialised professions again showed a significant correlation with PHSS. In comparison to model 5, the significant effects of EMPATHIC-30 full-scale scores, caregiver age, and room size disappeared. The fixed effect of infant sex became significant. Furthermore, the effect of siblings was significant. Additionally, the Aristotle score was a significant fixed effect, as well as the effect of parental role (mothers). The living distance from heart centre in km was significant in this model, and finally, the duration of stay at PCICU in days. This model explained approximately 46% of the variance (adjusted *R*^2^ = .46).
4. Model 8 (final model)
  In our final model 8 (investigating the subscale effect of EMPATHIC-30 information in subsample analyses), we found a significant intercept [*B* = 1.89, SE = 0.61, *t*(59) = 3.08, *p* = .003]. Similar to model 6, the EMPATHIC-30 subscale Information was significant [*B* = −0.20, SE = 0.09, *t*(59) = −2.29, *p* = .026], and the same significant effects as in model 7 were found. After mutual adjustment, female infants scored lower on PHSS than male infants [*B* = −0.34, SE = 0.13, *t*(59) = −2.59, *p* = .012]; similarly, infants with siblings scored lower on the PHSS [*B* = −0.44, SE = 0.14, *t*(59) = −3.12, *p* = .003]; a higher Aristotle score was associated with a higher PHSS score [*B* = 0.06, SE = 0.02, *t*(59) = 3.23, *p* = .002]; mothers rated their infants higher on PHSS than fathers [*B* = 0.69, SE = 0.28, *t*(59) = 2.47, *p* = .016]; infants of caregivers with highly complex, complex specialised and specialised professions scored higher than infants of caregivers with unskilled/semi-skilled professions [*B* = 1.05, SE = 0.24, *t*(59) = 4.39, *p* < .001; *B* = 0.54, SE = 0.24, *t*(59) = 2.24, *p* = .029; *B* = 0.61, SE = 0.20, *t*(59) = 3.10, *p* = .003, respectively]. A longer living distance from centre in km was associated with lower PHSS [*B* = −0.003, SE = 0.001, *t*(59) = −2.56, *p* = .013], and the duration of stay at PCICU in days was also associated with lower PHSS [*B* = −0.05, SE = 0.01, *t*(59) = −4.79, *p* < .001]. This model explained approximately 50% of the variance (adjusted *R*^2^ = .50). See Supplementary Table S3 for an overview of reported models, fixed effects and explained variance.

#### 3.3.2 Infant emotional health (EFS)

As before, the final, best fitting model is reported last. See Supplementary Table S4 for an overview of all fixed effects per model.

1. Model 5 (primary model)
  In model 5, the intercept was significant [*B* = 1.05, SE = 0.25, *t*(114) = 4.22, *p* < .001]. A higher infant age was associated with higher scores on the EFS [*B* = 0.003, SE = 0.0007, *t*(114) = 4.25, *p* < .001]. Furthermore, the fixed effect of siblings was significant [*B* = −0.49, SE = 0.14, *t*(114) = −3.45, *p* < .001], indicating that infants with siblings had lower EFS scores than infants without. Additionally, infants with genetic comorbidity scored lower on the EFS than infants without genetic comorbidity [*B* = −0.36, SE = 0.17, *t*(114) = −2.07, *p* = .040]. The parental professional level showed no significant effect. This model explained approximately 24% of the variance (adjusted *R*^2^ = .24).
2. Model 6
  Modelling individual EMPATHIC-30 subscales did not alter the results, see Supplementary Table S4.
3. Model 7
  In model 7, we found a significant intercept and comparable to model 5 infant age, siblings, genetic comorbidity and first-generation migration background were significant fixed effects. Additionally, prematurity became significant in this model, as well as the fixed effects of highly complex specialised, complex specialised and skilled professions, and the fixed effect of second-generation background. Finally, duration of stay at PCICU now contributed significantly. This model explained approximately 47% of the variance (adjusted *R*^2^ = .47).
4. Model 8 (final model)
  We conducted a final model 8 in order to check for EMPATHIC-30 subscales potentially obscuring the EMPATHIC-30 full-scale effect in model 7. The intercept was not significant [*B* = 0.93, SE = 0.62, *t*(57) = 1.49, *p* = .0142]. The EMPATHIC-30 subscale Care and Cure became significant [*B* = −0.38, SE = 0.15, *t*(57) = −2.51, *p* = .015], meaning that parents that gave higher ratings on this subscale scored their child lower on emotional symptoms. Additionally, the subscale Professional Attitude became significant [*B* = 0.39, SE = 0.18, *t*(57) = 2.14, *p* = .036]: more satisfaction with the professional attitude of the staff was associated with more emotional symptoms. Unlike in model 7, first generation migration background now only showed a trend [*B* = −1.07, SE = 0.55, *t*(57) = −1.93, *p* = .059]. All other effects were consistent with model 7: infants of caregivers with second generation migration background scored higher than infants of caregivers without migration background [*B* = 0.35, SE = 0.15, *t*(57) = 2.40, *p* = .020], a higher infant age was associated with higher scores on the EFS [*B* = 0.002, SE = 0.0008, *t*(57) = 3.04, *p* = .004], and the presence of siblings was associated with lower EFS scores [*B* = −0.67, SE = 0.17, *t*(57) = −4.06, *p* < .001]. Furthermore, prematurity was associated with lower EFS scores [*B* = −0.56, SE = 0.23, *t*(57) = −2.39, *p* = .020], as well as the presence of a genetic comorbidity [*B* = −0.50, SE = 0.18, *t*(57) = −2.85, *p* = .006]. Highly complex specialised professions [*B* = 1.43, SE = 0.29, *t*(57) = 5.01, *p* < .001], complex specialised professions [*B* = 1.16, SE = 0.26, *t*(57) = 4.40, *p* < .001], and specialised professions [*B* = 0.72, SE = 0.22, *t*(57) = 3.25, *p* = .002] were associated with higher EFS scores in comparison with unskilled/semiskilled professions, while the duration of PCICU stay was associated with lower EFS Scores [*B* = −0.04, SE = 0.01, *t*(57) = −2.90, *p* = .005]. This model explained approximately 50% of the variance (adjusted *R*^2^ = .50). See Supplementary Table S4 for an overview of reported models, fixed effects and explained variance.

## 4 Discussion

In this study, we investigated parent-rated infant physical and mental health during hospitalisation at two PCICUs in Germany and associations with parental satisfaction with FCC, various socio-demographic and medical factors. We used multilevel modelling with incremental complexity and repeated final models with added filters to achieve a subsample to reduce potential biases due to methodological challenges while conducting the study.

A higher parental satisfaction with FCC is associated with lower scores on the PHSS, reflecting better physical health. Based on multilevel models that include the five EMPATHIC-30 subscales for the whole group (model 6) and subsample (model 8), this effect is primarily driven by the Information subscale, while all other EMPATHIC-30 subscales were found to be non-significant. Accordingly, parents who are more satisfied with the information they receive from the health care team rated their infants as having better physical health. This key finding supports the important goal of FCC in facilitating caregivers' understanding of the medical situations. Higher satisfaction with the information received may relate to both the quantity and quality of information and may strengthen the alliance between caregivers and health-care professionals, lead to more effective caregiving strategies, and ultimately result in better physical health for those infants (57). Higher satisfaction with the information received may also lead to a more positive evaluation of the infant's physical situation, e.g., through facilitated trust in the health-care team (58). Other alternative explanations may play a role, such as parental optimism influencing both satisfaction with FCC and the subjective experience of the infant's health. Additionally, a more dedicated health-care team (e.g., through better nurse-to-patient ratio) might improve communication with parents and enhance the quality of infant care.

Furthermore, the caregiver's professional status is associated with infant physical health across all multilevel models. Infants of caregivers with highly complex specialised professions score lower in physical health than those of caregivers with unskilled or semi-skilled professions. This also holds for the filtered subsample, specifically for caregivers with highly complex specialised, complex specialised and specialised professions. This finding suggests a robust effect of professional level on the subjectively rated physical health of the infant. The professional level may be linked to adaptive (e.g., cognitive reappraisal, reframing) vs. maladaptive (e.g., self-distraction, behavioural disengagement) coping styles, thereby impacting perceptions of infant's health status (59). Supporting this, a Korean study investigated the coping strategies of 141 mothers whose infants were hospitalised at a neonatal intensive care unit (60). Results showed that higher educational levels and more social support were associated with more adaptive coping styles.

Above that, we found three effects in the full sample that disappeared in the methodologically stricter sub-sample analysis, which warrants further exploration of these associations in future studies: A higher caregiver age is associated with fewer reported physical symptoms, a first-generation migration background is associated with fewer physical symptoms and finally, a higher proportion of nights spent in a single room vs. in a room shared with other patients is linked to more physical symptoms.

Conversely, our subsample analyses revealed new significant fixed effects. Mothers rated their infants higher on physical symptom burden than fathers. One possibility is that mothers of very critically ill infants were more frequently visiting the infant during hospitalisation and thus filling out the questionnaires as main visitor more frequently. Alternatively, mothers may assess their infant's health more critically. In our sample, mothers were the primary caregiver in about 74%. As such, they are engaged in the infant's physical care, which may heighten their awareness of the infants' signals and health status. In relation to this, previous studies have shown that mothers of children with CHD reported lower quality of life and physical health compared to fathers, as well as more caregiving time (61), This may suggest that mothers either assess the infant's symptoms more accurately due to spending more time with them, or that their assessments are influenced by their quality of life (62).

The Aristotle Complexity Score and duration of stay in PCICU are two medical variables that were negatively associated with the PHSS in subsample analyses. A higher complexity relates to a higher symptom burden, thereby substantiating the PHSS as an indicator of infant's objective health. A longer duration of stay in the PCICU is associated with lower PHSS score and, consequently, fewer reported symptoms. This relationship may be explained by the absence of an intermediary ward at one of the two clinics, which resulted in a substantially longer period of monitoring and physical recovery in the PCICU—nearly twice as many days on average.

Furthermore, infants with siblings were reported as having fewer physical symptoms, suggesting a potential protective effect of siblings for infant physical health. Alternatively, caregivers with other children may experience a lower emotional burden, and subsequently more positive evaluation of infant physical health. Indeed, caregivers with other children had lower depression rates in the same sample, which may relate to the subjective evaluation of the physical symptom burden (Ferentzi et al, under review). Female infants scored lower than male infants in our subsample analyses, indicating fewer physical symptoms, which may reflect a sex disparity with regard to adverse medical outcomes in infancy (63, 64). Finally, a greater living distance from the centre relates to fewer physical symptoms, which might be due to temporary housing arrangements near the clinic or on-site for the duration of hospitalisation, and accordingly, less stress, more time spent with the infant, and/or better communication with the health care team.

Concerning infant emotional health, we found a robust effect for the presence of siblings in both the whole sample and the subsample, in that infants with siblings are rated lower on emotional symptoms than infants without siblings. This aligns with the findings on the PHSS subscale and may be similarly explained. Furthermore, in both the full-sample analysis and the sub-sample analysis, infants with genetic syndromes are rated lower on the EFS, indicating a lower emotional symptom burden, which may be explained by different temperamental profiles of children with and without genetic syndromes (65). We found a significant association between emotional functioning and infant age across all EFS models—for the whole group and the subgroup –, in that a higher infant age is associated with lower emotional functioning. This potentially points to age-dependent increases in observable infant behaviours, e.g., of crying and smiling, and this variability allowing for a more differentiated assessment of emotional health (66–68).

Furthermore, we found significant effects for subsample analyses only. Specifically, we found an association between two subscales of the EMPATHIC-30 (Professional Attitude, Care and Cure) and EFS scores, with opposing directions, which may explain the non-significant effect of the EMPATHIC-30 full-scale score in the preceding subsample model 7. While higher satisfaction with professional attitude is associated with a higher symptom burden, or lower emotional functioning, a higher satisfaction with Care and Cure is associated with a lower symptom burden, or higher emotional functioning. The subscale professional attitude reflects operational aspects (e.g., items “the team adhered to cleanliness and hygiene standards”, or “the staff respected our privacy as well as that of our infant/child”), which, in the context of COVID-19 restrictions, may relate to more restricted family access and consequently, less emotionally supportive care from healthcare professionals. By contrast, higher scores on Care and Cure (e.g., items “the doctors and nurses worked closely together”, or “the team watched our infant/our child carefully to prevent and treat pain”) may be indicative of more emotionally supportive care. Replication of our results due to the exploratory nature is vital to further disentangle different care aspects in relation to infant emotional health.

Moreover, infants of caregivers with a second-generation migration background experience a higher symptom burden than those of caregivers without migration background—a result not observed in the whole group analyses and that will need follow-up studies to explore potential explanations. Infants of parents with highly complex specialised professions, complex specialised and specialised professions show fewer emotional symptoms compared to infants of parents with unskilled professions. This pattern mirrors the associations found for physical health and may be explained in similar manner. Furthermore, we found that premature infants are rated higher on emotional functioning, which potentially may be explained by a longer adaption phase to the hospital environment due to care in the Neonatal Intensive Care Unit (NICU) by both infants and their caregivers. Finally, a longer duration of stay in the PCICU relates to a lower score on the EFS, similar to our finding on the PHSS, and may be similarly explained by the absence of an intermediary ward at one of the two clinics.

Our study has several methodological limitations. While parental proxy reports on infants' emotional and physical well-being are universally accepted and bias can be reduced as mentioned in the introduction, shared method variance remains a concern. The PEDSQL reflects parents' subjective experience of their infant's health over the past week. Reciprocal relationships between parental and child emotional well-being have been reported (62) and high anxiety states due to activation of the fight or flight response potentially lead to a focus on potential danger (69). There might thus be a common influence on both parent-rated child well-being and FCC rating. Confirmatory studies therefore benefit from more objective operationalisation of FCC and the inclusion of infant health as rated by healthcare professionals. Objective measurements of infant mental health could, for example, be physiological assessments (e.g., assessments of heart rate variability or cortisol levels) or behavioural observation (70–72). Furthermore, we included about 20 independent variables in a sample size of 305, which could pose potential for overfitting the model. Also, given the exploratory nature of the study, findings have to be interpreted with caution, and confirmatory follow-up studies are warranted. This is an important prerequisite for the substantiation of risk- and protective factors for infant physical and mental health in PCICUs, and to allow evidence-based development of FCC interventions. Finally, our study was observational and therefore no causality can be established. Intervention studies will be necessary to develop holistic care approaches that foster hospital environments that are protective rather than detrimental to infant mental health and development.

## 5 Conclusions

Our results show a unique combination of medical and contextual factors that explain a high amount of variance in infant physical health and emotional health outcomes. Interestingly, in the filtered subsample, the amount of explained variance increased substantially compared to the full-sample models. This means that while there was less variance due to the smaller sample size, a relatively higher proportion of variance was explained by variables retained in the model, with those variables relating more strongly and uniquely to PHSS and EFS. Furthermore, we found associations with objective medical parameters (e.g., the Aristotle complexity score), substantiating the PEDSQL as a valid measure of infant health. Generally, our results provide preliminary support for the idea that infants with CHD profit from a tailored approach to facilitating their physical and emotional health, accounting for the individual medical situation and unique constellation of environmental factors. Importantly, this is the very essence of family-centred care interventions: tailoring the hospital experience to the individual needs of the infant and the family system. Confirmatory studies are needed to support our findings.

## Data Availability

The raw data supporting the conclusions of this article will be made available by the authors, without undue reservation.
