# Supplementary material for: Indicators of physical and emotional health in infants during hospitalization in paediatric cardiac intensive care units
Source: Front Cardiovasc Med. 2026 Aug 13;13:1884496. doi: 10.3389/fcvm.2026.1884496 (PMC13519917; doi:10.3389/fcvm.2026.1884496)
Supplement: Supplementary file 1 [file Table1.docx]

| Table 1. Consecutive Multilevel Models for PEDSQL Physical Health. | | | | | | | | | | | | |
| --- | --- | --- | --- | --- | --- | --- | --- | --- | --- | --- | --- | --- |
| Model | Model Fit Statistics | | | | Model Comparisons Statistics | | | Fixed Effect Statistics | | | | |
| Model | Number of Parameters | AIC | BIC | Log-Likelihood | Chi^2^ | df | p | Fixed Effects | Estimate | Standard Error | T | P |
| 1 | 3 | 369.4 | 378.6 | -181.7 | - | - | - | Intercept | 1.55 | 0.06 | 25.49 | **<0.001*** |
| 2 | 4 | 363.4 | 375.7 | -177.7 | 7.96 | 1 | **0.005*** | Intercept | 2.90 | 0.47 | 6.10 | **<0.001*** |
|  |  |  |  |  |  |  |  | Empathic Total | -0.26 | 0.09 | -2.86 | **0.005*** |
| 3 | 33 | 369.7 | 470.7 | -151.8 | 51.77 | 29 | **0.006*** | Intercept | 3.16 | 0.77 | 4.09 | **<0.001*** |
|  |  |  |  |  |  |  |  | Empathic Total | -0.18 | 0.09 | -2.06 | **0.041*** |
|  |  |  |  |  |  |  |  | Child age in days | -0.0004 | 0.0006 | -0.67 | 0.504 |
|  |  |  |  |  |  |  |  | Child sex | 0.004 | 0.11 | 0.04 | 0.968 |
|  |  |  |  |  |  |  |  | Siblings | -0.25 | 0.12 | -2.05 | **0.042*** |
|  |  |  |  |  |  |  |  | Prematurity | -0.07 | 0.16 | -0.46 | 0.644 |
|  |  |  |  |  |  |  |  | Genetic comorbidity | -0.08 | 0.14 | -0.57 | 0.568 |
|  |  |  |  |  |  |  |  | Aristotle score | 0.01 | 0.02 | 0.84 | 0.400 |
|  |  |  |  |  |  |  |  | Caregiver age | -0.02 | 0.01 | -1.80 | 0.074 |
|  |  |  |  |  |  |  |  | Participant 1 (mother) | 0.22 | 0.25 | 0.90 | 0.370 |
|  |  |  |  |  |  |  |  | Participant 2 (both parents) | 0.49 | 0.36 | 1.34 | 0.181 |
|  |  |  |  |  |  |  |  | Legal status (single) | -0.17 | 0.12 | -1.39 | 0.166 |
|  |  |  |  |  |  |  |  | Legal status (divorced) | 0.19 | 0.32 | 0.58 | 0.561 |
|  |  |  |  |  |  |  |  | Living with partner | -0.41 | 0.24 | -1.68 | 0.095 |
|  |  |  |  |  |  |  |  | School 2 (middle to high) | -0.09 | 0.19 | -0.46 | 0.646 |
|  |  |  |  |  |  |  |  | School 3 (high) | -0.06 | 0.20 | -0.29 | 0.772 |
|  |  |  |  |  |  |  |  | Profession 2 (specialized) | 0.37 | 0.21 | 1.78 | 0.077 |
|  |  |  |  |  |  |  |  | Profession 3 (complex specialized) | 0.36 | 0.23 | 1.54 | 0.125 |
|  |  |  |  |  |  |  |  | Profession 4 (highly complex specialized) | 0.86 | 0.25 | 3.49 | **<0.001*** |
|  |  |  |  |  |  |  |  | Profession 5 (other) | -0.09 | 0.40 | -0.22 | 0.824 |
|  |  |  |  |  |  |  |  | Occupation 2 (part-time) | -0.27 | 0.18 | -1.49 | 0.137 |
|  |  |  |  |  |  |  |  | Occupation 4 (marginal) | 0.15 | 0.42 | 0.36 | 0.723 |
|  |  |  |  |  |  |  |  | Occupation 10 (on leave) | 0.08 | 0.13 | 0.62 | 0.535 |
|  |  |  |  |  |  |  |  | Occupation 11 (none) | 0.08 | 0.37 | 0.22 | 0.828 |
|  |  |  |  |  |  |  |  | Migration Background 1 (second gen) | -0.02 | 0.13 | -0.13 | 0.900 |
|  |  |  |  |  |  |  |  | Migration Background 2 (first gen) | -1.24 | 0.56 | -2.24 | **0.027*** |
|  |  |  |  |  |  |  |  | Language 2 (German first, second other) | 0.20 | 0.20 | -1.02 | 0.309 |
|  |  |  |  |  |  |  |  | Language 3 (other first, German second) | -0.10 | 0.24 | -0.40 | 0.690 |
|  |  |  |  |  |  |  |  | Distance from center in km | -0.0009 | 0.0006 | -1.70 | 0.092 |
|  |  |  |  |  |  |  |  | Room size PCICU | 0.27 | 0.17 | 1.56 | 0.120 |
|  |  |  |  |  |  |  |  | Duration of stay PCICU | -0.008 | 0.01 | -0.81 | 0.420 |
|  |  |  |  |  |  |  |  |  |  |  |  |  |
| 4 | 34 | 375.4 | 479.6 | -153.7 | 0.00 | 1 | 1.000 | Intercept | 2.09 | 0.56 | 3.74 | **<0.001*** |
|  |  |  |  |  |  |  |  | Child age in days | -0.0004 | 0.0006 | -0.62 | 0.534 |
|  |  |  |  |  |  |  |  | Child sex | 0.003 | 0.11 | 0.03 | 0.979 |
|  |  |  |  |  |  |  |  | Siblings | -0.29 | 0.12 | -2.40 | **0.018*** |
|  |  |  |  |  |  |  |  | Prematurity | -0.07 | 0.16 | -0.45 | 0.652 |
|  |  |  |  |  |  |  |  | Genetic comorbidity | -0.07 | 0.14 | -0.51 | 0.612 |
|  |  |  |  |  |  |  |  | Aristotle score | 0.02 | 0.02 | 0.93 | 0.356 |
|  |  |  |  |  |  |  |  | Caregiver age | -0.02 | 0.01 | -1.84 | 0.067 |
|  |  |  |  |  |  |  |  | Participant 1 (mother) | 0.24 | 0.25 | 0.93 | 0.353 |
|  |  |  |  |  |  |  |  | Participant 2 (both parents) | 0.53 | 0.37 | 1.44 | 0.151 |
|  |  |  |  |  |  |  |  | Legal status (single) | -0.15 | 0.12 | -1.20 | 0.233 |
|  |  |  |  |  |  |  |  | Legal status (divorced) | 0.17 | 0.32 | 0.52 | 0.607 |
|  |  |  |  |  |  |  |  | Living with partner | -0.39 | 0.24 | -1.60 | 0.112 |
|  |  |  |  |  |  |  |  | School 2 (middle to high) | -0.09 | 0.19 | -0.45 | 0.654 |
|  |  |  |  |  |  |  |  | School 3 (high) | -0.03 | 0.20 | -0.14 | 0.887 |
|  |  |  |  |  |  |  |  | Profession 2 (specialized) | 0.42 | 0.21 | 2.00 | **0.047*** |
|  |  |  |  |  |  |  |  | Profession 3 (complex specialized) | 0.42 | 0.24 | 1.78 | 0.077 |
|  |  |  |  |  |  |  |  | Profession 4 (highly complex specialized) | 0.89 | 0.25 | 3.58 | **<0.001*** |
|  |  |  |  |  |  |  |  | Profession 5 (other) | -0.03 | 0.40 | -0.08 | 0.940 |
|  |  |  |  |  |  |  |  | Occupation 2 (part-time) | -0.26 | 0.18 | -1.41 | 0.161 |
|  |  |  |  |  |  |  |  | Occupation 4 (marginal) | 0.19 | 0.42 | 0.44 | 0.659 |
|  |  |  |  |  |  |  |  | Occupation 10 (on leave) | 0.09 | 0.13 | 0.68 | 0.496 |
|  |  |  |  |  |  |  |  | Occupation 11 (none) | 0.16 | 0.38 | 0.43 | 0.672 |
|  |  |  |  |  |  |  |  | Migration Background 1 (second gen) | -0.0007 | 0.13 | -0.01 | 0.995 |
|  |  |  |  |  |  |  |  | Migration Background 2 (first gen) | -1.26 | 0.56 | -2.25 | **0.026*** |
|  |  |  |  |  |  |  |  | Language 2 (German first, second other) | -0.25 | 0.20 | -1.25 | 0.212 |
|  |  |  |  |  |  |  |  | Language 3 (other first, German second) | -0.07 | 0.24 | -0.27 | 0.785 |
|  |  |  |  |  |  |  |  | Distance from center in km | -0.001 | 0.0006 | -1.74 | 0.084 |
|  |  |  |  |  |  |  |  | Room size PCICU | 0.26 | 0.17 | 1.48 | 0.140 |
|  |  |  |  |  |  |  |  | Duration of stay PCICU | -0.008 | 0.01 | -0.82 | 0.411 |
|  |  |  |  |  |  |  |  |  |  |  |  |  |
| 5 | 369.7 | 470.7 | -151.8 |  |  |  |  | Intercept | 3.32 | 0.57 | 5.85 | **<0.001*** |
|  |  |  |  |  |  |  |  | Empathic Total | -0.25 | 0.09 | -2.93 | **0.004*** |
|  |  |  |  |  |  |  |  | Caregiver age | -0.02 | 0.01 | -2.51 | **0.013*** |
|  |  |  |  |  |  |  |  | Profession 2 (specialized) | 0.26 | 0.20 | 1.29 | 0.198 |
|  |  |  |  |  |  |  |  | Profession 3 (complex specialized) | 0.34 | 0.24 | 1.44 | **0.153** |
|  |  |  |  |  |  |  |  | Profession 4 (highly complex specialized) | 0.87 | 0.24 | 3.63 | **<0.001*** |
|  |  |  |  |  |  |  |  | Profession 5 (other) | -0.14 | 0.40 | -0.35 | 0.729 |
|  |  |  |  |  |  |  |  | Migration Background 1 (second gen) | -0.06 | 0.12 | -0.46 | 0.646 |
|  |  |  |  |  |  |  |  | Migration Background 2 (first gen) | -1.25 | 0.51 | -2.47 | **0.015*** |
|  |  |  |  |  |  |  |  | Room size PCICU | 0.36 | 0.18 | 2.01 | **0.046*** |
| 6 |  |  |  |  |  |  |  | Intercept | 2.92 | 0.56 | 5.21 | **<0.001*** |
|  |  |  |  |  |  |  |  | Empathic Information | -0.25 | 0.08 | -3.23 | **0.002*** |
|  |  |  |  |  |  |  |  | Caregiver age | -0.03 | 0.01 | -2.78 | **0.006*** |
|  |  |  |  |  |  |  |  | Participant 1 (mother) | 0.45 | 0.24 | 1.85 | 0.066 |
|  |  |  |  |  |  |  |  | Participant 2 (both parents) | 0.94 | 0.37 | 2.51 | **0.013*** |
|  |  |  |  |  |  |  |  | Profession 2 (specialized) | 0.28 | 0.20 | 1.38 | 0.170 |
|  |  |  |  |  |  |  |  | Profession 3 (complex specialized) | 0.45 | 0.24 | 1.90 | 0.059 |
|  |  |  |  |  |  |  |  | Profession 4 (highly complex specialized) | 0.87 | 0.24 | 3.63 | **<0.001*** |
|  |  |  |  |  |  |  |  | Profession 5 (other) | -0.14 | 0.40 | -0.35 | 0.728 |
| 7 |  |  |  |  |  |  |  | Intercept | 0.84 | 0.42 | 2.00 | 0.050 |
|  |  |  |  |  |  |  |  | Child sex | -0.28 | 0.13 | -2.11 | **0.039*** |
|  |  |  |  |  |  |  |  | Siblings | -0.54 | 0.14 | -3.97 | **<0.001*** |
|  |  |  |  |  |  |  |  | Aristotle score | 0.07 | 0.02 | 3.64 | **<0.001*** |
|  |  |  |  |  |  |  |  | Participant 1 (mother) | 0.66 | 0.29 | 2.30 | **0.025*** |
|  |  |  |  |  |  |  |  | Profession 2 (specialized) | 0.61 | 0.20 | 3.01 | **0.004*** |
|  |  |  |  |  |  |  |  | Profession 3 (complex specialized) | 0.49 | 0.25 | 1.98 | 0.052 |
|  |  |  |  |  |  |  |  | Profession 4 (highly complex specialized) | 1.04 | 0.25 | 4.20 | **<0.001*** |
|  |  |  |  |  |  |  |  | Profession 5 (other) | 1.02 | 0.58 | 1.175 | 0.086 |
|  |  |  |  |  |  |  |  | Distance from center in km | -0.003 | 0.001 | -2.75 | **0.008*** |
|  |  |  |  |  |  |  |  | Duration of stay PCICU | -0.05 | 0.01 | -4.51 | **<0.001*** |
| 8 |  |  |  |  |  |  |  | Intercept | 1.89 | 0.61 | 3.08 | **0.003*** |
|  |  |  |  |  |  |  |  | Empathic Information | -0.20 | 0.09 | -2.29 | **0.026*** |
|  |  |  |  |  |  |  |  | Child sex | -0.34 | 0.13 | -2.59 | **0.012*** |
|  |  |  |  |  |  |  |  | Siblings | -0.44 | 0.14 | -3.12 | **0.003*** |
|  |  |  |  |  |  |  |  | Aristotle score | 0.06 | 0.02 | 3.23 | **0.002*** |
|  |  |  |  |  |  |  |  | Participant 1 (mother) | 0.69 | 0.28 | 2.47 | **0.016*** |
|  |  |  |  |  |  |  |  | Profession 2 (specialized) | 0.61 | 0.20 | 3.10 | **0.003*** |
|  |  |  |  |  |  |  |  | Profession 3 (complex specialized) | 0.54 | 0.24 | 2.24 | **0.029*** |
|  |  |  |  |  |  |  |  | Profession 4 (highly complex specialized) | 1.05 | 0.24 | 4.39 | **<0.001*** |
|  |  |  |  |  |  |  |  | Profession 5 (other) | 0.83 | 0.57 | 1.45 | 0.154 |
|  |  |  |  |  |  |  |  | Distance from center in km | -0.002 | 0.001 | -2.56 | **0.013*** |
|  |  |  |  |  |  |  |  | Duration of stay PCICU | -0.05 | 0.01 | -4.79 | **<0.001*** |

| Table 2. Consecutive Multilevel Models for PEDSQL Emotion. | | | | | | | | | | | | |
| --- | --- | --- | --- | --- | --- | --- | --- | --- | --- | --- | --- | --- |
| Model | Model Fit Statistics | | | | Model Comparisons Statistics | | | Fixed Effect Statistics | | | | |
| Model | Number of Parameters | AIC | BIC | Log-Likelihood | Chi^2^ | df | p | Fixed Effects | Estimate | Standard Error | T | P |
| 1 | 3 | 309.5 | 317.9 | -151.7 | - | - | - | Intercept | 1.23 | 0.12 | 9.92 | **0.011*** |
| 2 | 4 | 305.5 | 316.7 | -148.8 | 5.94 | 1 | **0.015*** | Intercept | 2.62 | 0.58 | 4.54 | **<0.001*** |
|  |  |  |  |  |  |  |  | Empathic Total | -0.26 | 0.11 | -2.47 | **0.015*** |
| 3 | 33 | 299.6 | 392.1 | -116.8 | 63.95 | 29 | **<0.001*** | Intercept | 3.79 | 0.89 | 4.25 | **<0.001*** |
|  |  |  |  |  |  |  |  | Empathic Total | -0.21 | 0.10 | -2.08 | **0.040*** |
|  |  |  |  |  |  |  |  | Child age in days | 0.002 | 0.0007 | 3.17 | **0.002*** |
|  |  |  |  |  |  |  |  | Child sex | -0.19 | 0.14 | -1.42 | 0.158 |
|  |  |  |  |  |  |  |  | Siblings | -0.39 | 0.15 | -2.63 | **0.010*** |
|  |  |  |  |  |  |  |  | Prematurity | -0.26 | 0.20 | -1.27 | 0.205 |
|  |  |  |  |  |  |  |  | Genetic comorbidity | 0.30 | 0.16 | -1.87 | 0.064 |
|  |  |  |  |  |  |  |  | Aristotle score | -0.04 | 0.019 | -2.10 | **0.038*** |
|  |  |  |  |  |  |  |  | Caregiver age | -0.01 | 0.01 | -0.95 | 0.346 |
|  |  |  |  |  |  |  |  | Participant 1 (mother) | -0.39 | 0.30 | -1.29 | 0.199 |
|  |  |  |  |  |  |  |  | Participant 2 (both parents) | -0.13 | 0.44 | -0.29 | 0.775 |
|  |  |  |  |  |  |  |  | Legal status (single) | 0.09 | 0.14 | 0.66 | 0.510 |
|  |  |  |  |  |  |  |  | Legal status (divorced) | 0.49 | 0.37 | 1.31 | 0.192 |
|  |  |  |  |  |  |  |  | Living with partner | 0.09 | 0.25 | 0.38 | 0.705 |
|  |  |  |  |  |  |  |  | School 2 (middle to high) | -0.17 | 0.24 | -0.71 | 0.476 |
|  |  |  |  |  |  |  |  | School 3 (high) | -0.34 | 0.25 | -1.35 | 0.179 |
|  |  |  |  |  |  |  |  | Profession 2 (specialized) | -0.09 | 0.23 | -0.39 | 0.700 |
|  |  |  |  |  |  |  |  | Profession 3 (complex specialized) | 0.41 | 0.26 | 1.56 | 0.122 |
|  |  |  |  |  |  |  |  | Profession 4 (highly complex specialized) | 0.53 | 0.30 | 1.79 | 0.076 |
|  |  |  |  |  |  |  |  | Profession 5 (other) | -0.81 | 0.45 | -1.81 | 0.073 |
|  |  |  |  |  |  |  |  | Occupation 2 (part-time) | 0.07 | 0.23 | 0.30 | 0.762 |
|  |  |  |  |  |  |  |  | Occupation 4 (marginal) | -0.16 | 0.44 | -0.37 | 0.715 |
|  |  |  |  |  |  |  |  | Occupation 10 (on leave) | 0.10 | 0.15 | 0.66 | 0.514 |
|  |  |  |  |  |  |  |  | Occupation 11 (none) | -0.57 | 0.38 | -1.52 | 0.131 |
|  |  |  |  |  |  |  |  | Migration Background 1 (second gen) | -0.06 | 0.16 | -0.35 | 0.724 |
|  |  |  |  |  |  |  |  | Migration Background 2 (first gen) | -1.42 | 0.60 | -2.38 | **0.019*** |
|  |  |  |  |  |  |  |  | Language 2 (German first, second other) | 0.02 | 0.21 | 0.07 | 0.944 |
|  |  |  |  |  |  |  |  | Language 3 (other first, German second) | 0.10 | 0.32 | 0.30 | 0.762 |
|  |  |  |  |  |  |  |  | Distance from center in km | -0.0006 | 0.0006 | -0.99 | 0.326 |
|  |  |  |  |  |  |  |  | Room size PCICU | -0.04 | 0.20 | -0.23 | 0.82 |
|  |  |  |  |  |  |  |  | Duration of stay PCICU | -0.02 | 0.01 | -1.49 | 0.141 |
|  |  |  |  |  |  |  |  |  |  |  |  |  |
| 4 | 34 | 304.1 | 399.4 | -118.0 | 0.00 | 1 | 1.000 | Intercept | 2.88 | 0.66 | 4.37 | **<0.001*** |
|  |  |  |  |  |  |  |  | Child age in days | 0.002 | 0.0007 | 3.06 | **0.003*** |
|  |  |  |  |  |  |  |  | Child sex | -0.18 | 0.14 | -1.35 | 0.179 |
|  |  |  |  |  |  |  |  | Siblings | -0.44 | 0.14 | -3.10 | **0.002*** |
|  |  |  |  |  |  |  |  | Prematurity | -0.26 | 0.20 | -1.29 | 0.199 |
|  |  |  |  |  |  |  |  | Genetic comorbidity | -0.28 | 0.16 | -1.70 | 0.093 |
|  |  |  |  |  |  |  |  | Aristotle score | -0.04 | 0.02 | -1.89 | 0.062 |
|  |  |  |  |  |  |  |  | Caregiver age | -0.009 | 0.01 | -0.86 | 0.392 |
|  |  |  |  |  |  |  |  | Participant 1 (mother) | -0.44 | 0.30 | -1.47 | 0.145 |
|  |  |  |  |  |  |  |  | Participant 2 (both parents) | -0.22 | 0.44 | -0.51 | 0.614 |
|  |  |  |  |  |  |  |  | Legal status (single) | 0.11 | 0.14 | 0.77 | 0.445 |
|  |  |  |  |  |  |  |  | Legal status (divorced) | 0.52 | 0.38 | 1.38 | 0.171 |
|  |  |  |  |  |  |  |  | Living with partner | 0.11 | 0.25 | 0.44 | 0.661 |
|  |  |  |  |  |  |  |  | School 2 (middle to high) | -0.14 | 0.24 | -0.61 | 0.541 |
|  |  |  |  |  |  |  |  | School 3 (high) | -0.29 | 0.25 | -1.15 | 0.253 |
|  |  |  |  |  |  |  |  | Profession 2 (specialized) | -0.12 | 0.23 | -0.52 | 0.604 |
|  |  |  |  |  |  |  |  | Profession 3 (complex specialized) | 0.38 | 0.26 | 1.47 | 0.143 |
|  |  |  |  |  |  |  |  | Profession 4 (highly complex specialized) | 0.49 | 0.30 | 1.64 | 0.104 |
|  |  |  |  |  |  |  |  | Profession 5 (other) | -0.76 | 0.45 | -1.71 | 0.090 |
|  |  |  |  |  |  |  |  | Occupation 2 (part-time) | 0.04 | 0.23 | 0.17 | 0.867 |
|  |  |  |  |  |  |  |  | Occupation 4 (marginal) | -0.25 | 0.45 | -0.55 | 0.582 |
|  |  |  |  |  |  |  |  | Occupation 10 (on leave) | 0.09 | 0.15 | 0.60 | 0.551 |
|  |  |  |  |  |  |  |  | Occupation 11 (none) | -0.64 | 0.37 | -1.70 | 0.092 |
|  |  |  |  |  |  |  |  | Migration Background 1 (second gen) | -0.04 | 0.16 | -0.25 | 0.803 |
|  |  |  |  |  |  |  |  | Migration Background 2 (first gen) | -1.55 | 0.61 | -2.56 | **0.012*** |
|  |  |  |  |  |  |  |  | Language 2 (German first, second other) | -0.04 | 0.21 | -0.19 | 0.849 |
|  |  |  |  |  |  |  |  | Language 3 (other first, German second) | 0.24 | 0.32 | 0.75 | 0.457 |
|  |  |  |  |  |  |  |  | Distance from center in km | -0.0006 | 0.0006 | -1.03 | 0.304 |
|  |  |  |  |  |  |  |  | Room size PCICU | -0.02 | 0.20 | -0.10 | 0.919 |
|  |  |  |  |  |  |  |  | Duration of stay PCICU | -0.02 | 0.01 | -1.64 | 0.104 |
|  |  |  |  |  |  |  |  |  |  |  |  |  |
| 5 |  | 301.6 | 396.9 | -116.8 |  |  |  | Intercept | 1.05 | 0.25 | 4.22 | **<0.001*** |
|  |  |  |  |  |  |  |  | Child age in days | 0.003 | 0.0007 | 4.25 | **<0.001*** |
|  |  |  |  |  |  |  |  | Siblings | -0.49 | 0.14 | -3.45 | **<0.001*** |
|  |  |  |  |  |  |  |  | Genetic comorbidity | -0.36 | 0.17 | -2.07 | **0.040*** |
|  |  |  |  |  |  |  |  | Profession 2 (specialized) | -0.003 | 0.243 | -0.01 | 0.991 |
|  |  |  |  |  |  |  |  | Profession 3 (complex specialized) | 0.42 | 0.28 | 1.50 | 0.136 |
|  |  |  |  |  |  |  |  | Profession 4 (highly complex specialized) | 0.50 | 0.29 | 1.71 | 0.089 |
|  |  |  |  |  |  |  |  | Profession 5 (other) | -0.65 | 0.48 | -1.35 | 0.181 |
| 6 |  |  |  |  |  |  |  | Intercept | 1.05 | 0.25 | 4.22 | **<0.001*** |
|  |  |  |  |  |  |  |  | Child age in days | 0.003 | 0.0007 | 4.25 | **<0.001*** |
|  |  |  |  |  |  |  |  | Siblings | -0.49 | 0.14 | -3.45 | **<0.001*** |
|  |  |  |  |  |  |  |  | Genetic comorbidity | -0.36 | 0.17 | -2.07 | **0.040*** |
|  |  |  |  |  |  |  |  | Profession 2 (specialized) | -0.003 | 0.24 | -0.01 | 0.991 |
|  |  |  |  |  |  |  |  | Profession 3 (complex specialized) | 0.42 | 0.28 | 1.50 | 0.136 |
|  |  |  |  |  |  |  |  | Profession 4 (highly complex specialized) | 0.50 | 0.29 | 1.71 | 0.089 |
|  |  |  |  |  |  |  |  | Profession 5 (other) | -0.65 | 0.48 | -1.34 | 0.181 |
| 7 |  |  |  |  |  |  |  | Intercept | 1.04 | 0.29 | 3.62 | **<0.001*** |
|  |  |  |  |  |  |  |  | Child age in days | 0.003 | 0.0008 | 3.05 | **0.004*** |
|  |  |  |  |  |  |  |  | Siblings | -0.69 | 0.16 | -4.34 | **<0.001*** |
|  |  |  |  |  |  |  |  | Prematurity | -0.60 | 0.24 | -2.48 | **0.017*** |
|  |  |  |  |  |  |  |  | Genetic comorbidity | -0.41 | 0.18 | -2.24 | **0.029*** |
|  |  |  |  |  |  |  |  | Profession 2 (specialized) | 0.67 | 0.23 | 2.96 | **0.004*** |
|  |  |  |  |  |  |  |  | Profession 3 (complex specialized) | 1.02 | 0.27 | 3.77 | **<0.001*** |
|  |  |  |  |  |  |  |  | Profession 4 (highly complex specialized) | 1.31 | 0.30 | 4.45 | **<0.001*** |
|  |  |  |  |  |  |  |  | Profession 5 (other) | 0.01 | 0.58 | 0.01 | 0.990 |
|  |  |  |  |  |  |  |  | Migration Background 1 (second gen) | 0.33 | 0.15 | 2.15 | **0.036*** |
|  |  |  |  |  |  |  |  | Migration Background 2 (first gen) | -1.27 | 0.57 | -2.23 | **0.030*** |
|  |  |  |  |  |  |  |  | Duration of stay PCICU | -0.04 | 0.01 | -2.66 | **0.010*** |
| 8 |  |  |  |  |  |  |  | Intercept | 0.93 | 0.62 | 1.49 | 0.142 |
|  |  |  |  |  |  |  |  | Empathic Professional Attitude | 0.39 | 0.18 | 2.14 | **0.036*** |
|  |  |  |  |  |  |  |  | Empathic Care and Cure | -0.38 | 0.15 | -2.51 | **0.015*** |
|  |  |  |  |  |  |  |  | Child age in days | 0.002 | 0.0008 | 3.04 | **0.004*** |
|  |  |  |  |  |  |  |  | Siblings | -0.67 | 0.17 | -4.06 | **<0.001*** |
|  |  |  |  |  |  |  |  | Prematurity | -0.56 | 0.23 | -2.39 | **0.020*** |
|  |  |  |  |  |  |  |  | Genetic comorbidity | -0.50 | 0.18 | -2.85 | **0.006*** |
|  |  |  |  |  |  |  |  | Profession 2 (specialized) | 0.72 | 0.22 | 3.25 | **0.002*** |
|  |  |  |  |  |  |  |  | Profession 3 (complex specialized) | 1.16 | 0.26 | 4.40 | **<0.001*** |
|  |  |  |  |  |  |  |  | Profession 4 (highly complex specialized) | 1.43 | 0.29 | 5.01 | **<0.001*** |
|  |  |  |  |  |  |  |  | Profession 5 (other) | 0.28 | 0.56 | 0.50 | 0.618 |
|  |  |  |  |  |  |  |  | Migration Background 1 (second gen) | 0.35 | 0.15 | 2.40 | **0.020*** |
|  |  |  |  |  |  |  |  | Migration Background 2 (first gen) | -1.07 | 0.55 | -1.93 | 0.059 |
|  |  |  |  |  |  |  |  | Duration of stay PCICU | -0.04 | 0.01 | -2.90 | **0.005*** |

| Table 3.  Reported multilevel models for Infant Physical Health (PHSS) | | | | | | | | |
| --- | --- | --- | --- | --- | --- | --- | --- | --- |
|  | M1 | M2 | M3 | M4 | M5 | M6 | M7 | M8 |
| Intercept | + | + | + | + | + | + | + | + |
| Empathic Full-Scale |  | - | - |  | - |  |  |  |
| Empathic-I (Information) |  |  |  |  |  | - |  | - |
| Empathic-CC (Care and Cure) |  |  |  |  |  |  |  |  |
| Empathic-PP (Parental Participation) |  |  |  |  |  |  |  |  |
| Empathic-O (Organisation) |  |  |  |  |  |  |  |  |
| Empathic-PA (Professional Attitude) |  |  |  |  |  |  |  |  |
| Child Age |  |  | NS | NS |  |  |  |  |
| Child Sex |  |  | NS | NS |  |  | - | - |
| Siblings |  |  | - | - |  |  | - | - |
| Prematurity |  |  | NS | NS |  |  |  |  |
| Genetic Comorbidity |  |  | NS | NS |  |  |  |  |
| Aristotle Score |  |  | NS | NS |  |  | + | + |
| Caregiver Age |  |  | - | - | - | - |  |  |
| Parental Role (mother) |  |  | NS | NS |  | + | + | + |
| Parental Role (father) |  |  | NS | NS |  | + |  |  |
| Legal Status (single) |  |  | NS | NS |  |  |  |  |
| Legal Status (divorced) |  |  | NS | NS |  |  |  |  |
| Living with Partner (yes) |  |  | NS | NS |  |  |  |  |
| School Education (high) |  |  | NS | NS |  |  |  |  |
| School Education (middle to high) |  |  | NS | NS |  |  |  |  |
| Prof Level (specialised) |  |  | + | + | NS | NS | + | + |
| Prof Level (complex specialised) |  |  | NS | + | NS | + | + | + |
| Prof Level (highly complex specialised) |  |  | + | + | + | + | + | + |
| Prof Level (uncategorised) |  |  | NS | NS | NS | NS | + | NS |
| Occupational Situation (part-time) |  |  | NS | NS |  |  |  |  |
| Occupational Situation (marginal employment) |  |  | NS | NS |  |  |  |  |
| Occupational Situation (on leave) |  |  | NS | NS |  |  |  |  |
| Occupational Situation (none) |  |  | NS | NS |  |  |  |  |
| Migration Background (second generation) |  |  | NS | NS | NS |  |  |  |
| Migration Background (first generation) |  |  | - | - | - |  |  |  |
| Language (German first, second other) |  |  | NS | NS |  |  |  |  |
| Language (other first, second German) |  |  | NS | NS |  |  |  |  |
| Distance from Centre |  |  | NS | - |  |  | - | - |
| PCICU Stay |  |  | NS | NS |  |  | - | - |
| Room Size (Nights in single room / total number of nights) |  |  | NS | NS | + |  |  |  |
| Model Improvement / Adjusted R^2^ |  | ** | ** | NS | .16 | .16 | .46 | .50 |
| *Note: A higher PHSS score indicates more symptoms; Significant fixed effects in green and statistical trends in blue (+ indicating a positive association, - indicating a negative association), non-significant effects (indicated as NS) in orange.* | | | | | | | | |

| Table 4.  Reported multilevel models for Infant Emotional Health (EFS) | | | | | | | | |
| --- | --- | --- | --- | --- | --- | --- | --- | --- |
|  | **M1** | **M2** | **M3** | **M4** | **M5** | **M6** | **M7** | **M8** |
| Intercept | + | + | + | + | + | + | + | NS |
| Empathic Full-Scale |  | - | - |  |  |  |  |  |
| Empathic-I (Information) |  |  |  |  |  |  |  |  |
| Empathic-CC (Care and Cure) |  |  |  |  |  |  |  | - |
| Empathic-PP (Parental Participation) |  |  |  |  |  |  |  |  |
| Empathic-O (Organisation) |  |  |  |  |  |  |  |  |
| Empathic-PA (Professional Attitude) |  |  |  |  |  |  |  | + |
| Child Age |  |  | + | + | + | + | + | + |
| Child Sex |  |  | NS | NS |  |  |  |  |
| Siblings |  |  | - | - | - | - | - | - |
| Prematurity |  |  | NS | NS |  |  | - | - |
| Genetic Comorbidity |  |  | - | - | - | - | - | - |
| Aristotle Score |  |  | - | - |  |  |  |  |
| Caregiver Age |  |  | NS | NS |  |  |  |  |
| Parental Role (mother) |  |  | NS | NS |  |  |  |  |
| Parental Role (father) |  |  | NS | NS |  |  |  |  |
| Legal Status (single) |  |  | NS | NS |  |  |  |  |
| Legal Status (divorced) |  |  | NS | NS |  |  |  |  |
| Living with Partner (yes) |  |  | NS | NS |  |  |  |  |
| School Education (high) |  |  | NS | NS |  |  |  |  |
| School Education (middle to high) |  |  | NS | NS |  |  |  |  |
| Prof Level (specialised) |  |  | NS | NS | NS | NS | + | + |
| Prof Level (complex specialised) |  |  | NS | NS | NS | NS | + | + |
| Prof Level (highly complex specialised) |  |  | + | NS | + | + | + | + |
| Prof Level (uncategorised) |  |  | - | - | NS | NS | NS | NS |
| Occupational Situation (part-time) |  |  | NS | NS |  |  |  |  |
| Occupational Situation (marginal employment) |  |  | NS | NS |  |  |  |  |
| Occupational Situation (on leave) |  |  | NS | NS |  |  |  |  |
| Occupational Situation (none) |  |  | NS | - |  |  |  |  |
| Migration Background (second generation) |  |  | NS | NS |  |  | + | + |
| Migration Background (first generation) |  |  | - | - |  |  | - | - |
| Language (German first, second other) |  |  | NS | NS |  |  |  |  |
| Language (other first, second German) |  |  | NS | NS |  |  |  |  |
| Distance from Centre |  |  | NS | NS |  |  |  |  |
| PCICU Stay |  |  | NS | NS |  |  | - | - |
| Room Size |  |  | NS | NS |  |  |  |  |
| Model Improvement / Adjusted R^2^ |  | * | *** | NS | .24 | .24 | .47 | .50 |
| *Note: A higher EFS score indicates more symptoms; Significant fixed effects in green and statistical trends in blue (+ indicating a positive association, - indicating a negative association), non-significant effects (indicated as NS) in orange.* | | | | | | | | |
